# Computer-Based Cognitive Training vs. Paper-and-Pencil Training for Language and Cognitive Deficits in Greek Patients with Mild Alzheimer’s Disease: A Preliminary Study

**DOI:** 10.3390/healthcare11030443

**Published:** 2023-02-03

**Authors:** Eleni-Nefeli Georgopoulou, Anastasia Nousia, Vasileios Siokas, Maria Martzoukou, Elli Zoupa, Lambros Messinis, Efthimios Dardiotis, Grigorios Nasios

**Affiliations:** 1 Department of Speech and Language Therapy, School of Health Sciences, University of Ioannina, 45500 Ioannina, Greece; 2 Department of Neurology, Faculty of Medicine, School of Health Sciences, University of Thessaly, 41500 Larissa, Greece; 3 Larisa Day Care Center of People with Alzheimer’s Disease, Association for Regional Development and Mental Health (EPAPSY), 15124 Marousi, Greece; 4 Lab of Cognitive Neuroscience, School of Psychology, Aristotle University of Thessaloniki, 54124 Thessaloniki, Greece

**Keywords:** computer-based cognitive training, paper-pencil cognitive training, cognitive training, Alzheimer’s disease

## Abstract

The purpose of the present study was to explore whether Computer-Based Cognitive Training (C-BCT) versus Paper-Pencil Cognitive Training (P-PCT) is more beneficial in improving cognitive and language deficits in Greek patients living with Alzheimer’s disease (pwAD). Twenty pwAD were assigned to two groups: (a) the C-BCT group, receiving a computer-based cognitive training program using the RehaCom software, and (b) the P-PCT group, which received cognitive training using paper and pencil. The cognitive training programs lasted 15 weeks and were administered twice a week for approximately one hour per session. The analyses of each group’s baseline versus endpoint performance demonstrated that the P-PCT group improved on delayed memory, verbal fluency, attention, processing speed, executive function, general cognitive ability, and activities of daily living. In contrast, the C-BCT group improved on memory (delayed and working), naming, and processing speed. Comparisons between the two groups (C-BCT vs. P-PCT) revealed that both methods had significant effects on patients’ cognition, with the P-PCT method transferring the primary cognitive benefits to real-life activities. Our findings indicate that both methods are beneficial in attenuating cognitive and language deficits in pwAD. The need for large-scale neurobehavioral interventions to further clarify this issue, however, remains a priority.

## 1. Introduction

Alzheimer’s disease (AD) is the most prevalent form of dementia, deeply affecting patients at a plethora of levels, including cognitive and language skills [1,2,3]. Its etiology remains yet not fully understood, as genetic, environmental, and epigenetics factors appear to confer susceptibility to AD [4,5]. Unfortunately, the current AD pharmacological therapies have limited impact on the deceleration or reversion of disease progression [6,7]. Consequently, the research community is focusing on discovering effective interventions to supplement the current pharmacological treatments [8] and, furthermore, to improve the effective management of possible AD-precipitating factors [7,9]. Cognitive training appears to have beneficial effects on people living with AD (pwAD), since it may stabilize, decelerate, or even improve their cognitive decline [10].

When considering the issue of cognitive training for AD, the most popular and effective methodsare Computer-Based Cognitive Training (C-BCT) programs and the traditional, ecologically designed, Paper–Pencil Cognitive Training (P-PCT). In particular, several studies using C-BCT indicate that participants, after completing the intervention, perform better on global cognitive function [10,11,12,13,14,15], attention [10,13,15,16], memory (working, verbal, episodic, and objective memory), [10,13,15,16,17,18] language (verbal fluency, verbal expression, etc.) [10,12,13,15], and executive functions [10,12,15], and improvement is also noted on participants’ daily functions, and quality of life [14,15]. Although the aforementioned studies demonstrated improvement in several domains, there are indications of domains that remain stable. Specifically, no improvement has been reported on visual memory [15], global cognitive function [18], repetition [10,18], executive function [16], and verbal fluency [18]. Similarly, several studies have reported beneficial effects for groups trained using P-PCT in the domains of memory, attention, language, functional abilities, emotional level, and executive functions [12,17,19,20,21,22,23,24,25,26,27,28]. Ιt is worth mentioning at this point that not only these non-pharmacological cognitive approaches but also other multi-component non-pharmacological approaches such as physical exercise, [29] music therapy [30], and more recently transcranial magnetic stimulation combined with cognitive training [31] have been used and investigated for their benefits.

The question, however, of whether both methods (C-BCT versus P-PCT) are equally impactful for mild AD patients still stands. There are only three existing studies on individuals with Mild Cognitive Impairment (MCI), mild AD, and dementia which have compared the efficacious impact between C-BCT and P-PCT programs [14,17,26]. In particular, the recent study conducted by Tsolaki et al. (2017) on patients with multi-domain amnestic MCI evaluated the efficacy of a C-BCT versus an ecologically designed program of traditional cognitive training which was applied by P-PCT. They found that the C-BCT group, showed improvement on working memory and speed of switching attention, whereas the P-PCT group, showed improvement in general cognitive function, learning ability, delayed verbal recall, visual perception, visual memory, verbal fluency, and Instrumental Activities of Daily Living (IADL) [26]. Furthermore, the study of Lee, Yip, Yu and Man (2013) investigated the efficacy of computer-based training programs versus therapist-led cognitive training on individuals with mild AD, targeting the enhancement of memory and attention. The results showed an improvement for the group that attended the computer-based program, in that they presented a remarkable change in their general cognitive functions and daily functioning, while for the group that attended the therapist-led training program, significant improvements were noted on an emotional and functional level [14]. Lastly, the study of Man, Chung, and Lee (2012) examined the clinical efficacy of a memory training program delivered either by a computer-based or by a paper-and-pencil method on individuals with questionable dementia. The results indicated that both groups benefited from the intervention, but the group that had received a computer-based (virtual reality) program had better performance in more domains (objective memory and episodic information) compared to the group that attended the paper–pencil (subjective memory) training [17].

To the best of our knowledge, however, no study, until now, has investigated the efficacy of a comprehensive neuropsychological program (cognitive and language) of Greek pwAD. The aim of the present study, was to explore the efficacy of a specific cognitive training program, applied by C-BCT, and an ecologically designed, similar program of traditional cognitive training, applied by P-PCT to individuals with mild AD. A secondary aim was to evaluate the prospect of the cognitive benefits been transferred to everyday functioning capacity.

## 2. Method

### 2.1. Participants

**Figure 1:**
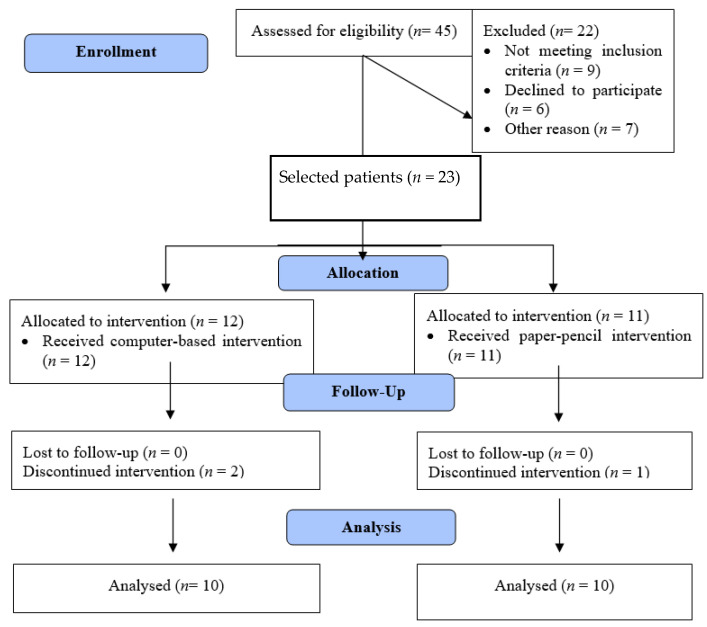
Participants’ flow diagram.

Over a period of six months (January to June 2018), a total of 45 patients with mild AD, native Greek language speakers, attended the Clinical Laboratory of Speech and Language Therapy of the University of Ioannina and were evaluated for their suitability of inclusion in the study. Twenty-two patients were excluded for various reasons (see Figure 1, flow diagram), resulting in 23 mild AD patients being enrolled in the study (initially, groups of 12 and 11 patients as the flow diagram indicates, of which 3 discontinued). The patients were assigned to two groups: (a) a C-BCT group, trained with the RehaCom software (3 males, 9 females), and (b) a P-PCT group (3 males, 8 females) which underwent the same cognitive intervention training, but by using paper-and-pencil training. Three of these patients dropped out during the period of training, so finally, the resulting 20 participants were analyzed (C-BCT; 3 males, 7 females, P-PCT; 3 males, 7 females).

The participants included in the study fulfilled the under mentioned criteria: (1)the patient diagnosis of AD was based on the National Institute of Neurological and Communicative Disorders and Stroke criteria and the Alzheimer’s and Related Disorders Association (NINCDS-ADRDA) criteria, (2) early-stage AD (Clinical Dementia Rating score (CDR) [32] of 1 and Montreal Cognitive Assessment (MoCA) [33,34] score of 16–21/30), (3) 60 to 80 years old, and (4) having completed at least 6 years of formal education. Patients were excluded from the study as follows: (1) presence of major psychiatric disorders (e.g., psychotic symptoms or disorders, alcohol or illegal drug abuse, depression, and ADHD), (2) presence of any other neurological disorder (e.g., traumatic brain injury, epilepsy, and stroke), and (3) visual/hearing impairment or writing/reading disability of sufficient degree to impact the performance in the assessment of this research. All patients had undergone clinical neurological assessment, blood tests, and brain magnetic resonance imaging scans and presented no evidence of any other diseases/impairments.

### 2.2. Procedure

#### 2.2.1. Neurological, Neuropsychological and Language Assessment

In order to assess the participants’ cognitive status (delayed and episodic memory, processing speed, executive function, attention, and recognition) and language abilities (recall, naming, and semantic fluency, domains which address performance deficits due to AD), the following tests were used: (a) MoCA [33,34], (b) Trail-Making Test, part A and part B (TMT A and TMT B) [35], (c) digit forward and backward tests [36], (d) repeat and delayed memory tests [37], (e) verbal fluency test [38], and (f) Boston Naming Test (BNT) [39]. In addition, the participants were assessed for depressive symptomatology by using the Geriatric Depression Scale (GDS), [40] whereas everyday functional activities were estimated with the Instrumental Activities of Daily Living (IADL) questionnaire [41]. The participants’ evaluation was performed both by a behavioral neurologist and by a highly experienced clinical neuropsychologist at baseline and after completing the intervention program. Considering the initial assessment phase, the neuropsychologist was unaware of the neurologist’s assessment and vice versa. Therefore, their assessment results for both groups did not affect either the process of the diagnosis or the patients’ selection when, finally, an individual speech therapist randomly divided the patients into the two groups (P-PCT and C-BCT).

#### 2.2.2. Measures and Variables Description

The following variables were considered for every participant, when possible, as they may confound the relationship between cognitive performances: age in years at the time of the first evaluation, education in years of formal schooling (as continuous variables), gender, and geriatric depression symptoms. Moreover, the descriptions of the measures are listed below.

MoCA—Montreal Cognitive assessment [34]. It is a brief, cognitive screening, 30-question test, in which the score ranges from 0 to 30. A score of 26 or higher is generally considered normal. The cognitive areas tested and the scoring breakdown areas follows: visuospatial and executive functioning, 5 points, animal naming, 3 points, attention, 6 points, language, 3 points, abstraction, 2 points, delayed recall (short-term memory), 5 points, orientation, 6 points. One additional point is added to the examinees’ score if (s)he has not attended 12 years of formal education.

The use of immediate word recall and delayed memory test [37] was chosen in order to evaluate delayed memory and recall. A list of 10 words is provided to the examinees, and they are asked to repeat as many as they can manage in every trial, for 4 consecutive trials. For the delayed memory task, after a period of 30 min, the participants are asked to recall as many words as they remember from the initial list (10 words).

BNT—Boston Naming Test [39]. The examinees are asked to name 15 line drawings of common or rarely seen objects in 20 s for each one. The maximum score is 15, awarding one point for every correct accurate answer.

Verbal Fluency task (semantic) [38]. Here, the participants are given 30 s to generate distinct words for each one of the following three categories: fruit, vegetables, and objects. The scores for all of the three categories are finally summed.

DSF (Digit Span Forward) and DSB (Digit Span Backward) test [36]. A verbal task, involving the oral presentation of spans of digits. The participants are requested to repeat the digits, either in the presented order (DSF), measuring verbal working memory and attention, or in reverse order (DSB), measuring cognitive control and executive function.

TMT-A and TMT-B—Trail consisting of tests A and B [35]. Both parts of the test consist of a set of 25 dots, filled with numbers (TMT-A) or with both numbers and letters (TMT-B), distributed over a sheet of paper. Part A examines processing speed, and Part B executive functioning. In TMT-A, the participant is requested to connect the targets with a line, in a sequential order. In TMT-B, with the targets including both numbers (1–13) and letters (A–L), the task taker also has to connect the dots in a sequential order, drawing a line, but this time alternating letters and numbers (as in 1-A-2-B-3-C, etc.). The examinee should finish both parts in the shortest time possible and without lifting the pencil/pen from the paper. If the patient fails to complete both parts in a period of 5 min, it is unnecessary to continue the test.

IADL—Instrumental Activities of Daily Living [41] scale, assessing a person’s ability to perform everyday living tasks such as using a telephone, doing laundry, and handling finances. It measures eight domains and could be administered in 10 to 15 min. The most common method is to rate each item either dichotomously (0 = less able, 1 = more able) or trichotomously (1 = unable, 2 = needs assistance, 3 = independent) and sum the scores of the eight responses. The higher the score, the greater the person’s function.

#### 2.2.3. Computer-Based Cognitive Training with RehaCom

The C-BCT group received 30 sessions on an individual basis, lasting 60 min each, for a total period of 15 weeks (i.e., approximately 2 sessions per week). The intervention was delivered by the RehaCom, a user-friendly cognitive software, widely used in Europe over the last years. The software provides a multi-module (over 20 different modules) cognitive rehabilitation option. The software program is also available in various languages, including Greek. The RehaCom provides the option of the use of a panel (specially designed), which can be combined with a large screen, making the training for elderly patients easier. Several cognitive domains were trained, including memory (episodic, delayed, verbal memory), executive functions, processing speed, and attention. The first level of training for the participants was the initial “beginner’s level” of the software, with automatic adaptation of the training modules tasks, based on the user’s performance. This provided the opportunity of adopting several difficulty levels and length of sessions depending on the patient’s performance. During the training preparation, the speech language therapist explained the RehaCom software to the participants. The researcher then helped them during the first attempt to familiarize with this software by providing some examples of each exercise. Continuous assistance was provided to the participants throughout the training period when difficulties arose (see Appendix A).

#### 2.2.4. Paper-and-Pencil Cognitive Training

For the P-PCT group, the content and the structure of the intervention program were similar to those of the program followed by the C-BCT group, except for the mode of delivery. The 30 sessions were run on an individual basis, lasting 60 min each for a total period of 15 weeks. Colored print, hard copy images were used. The speech language therapist, throughout the program, provided the same instruction and positive feedback to the participants, considering their individual needs. The difficulty level was also adjusted and recorded, meeting the abilities of the patients, and again replicating the tasks of the software per level.

The research protocol (for C-BCT and P-PCT) was approved by the Ethics Committee of the Medical School of Larisa, University of Thessaly, and was conducted in accordance with the Declaration of Helsinki’s principles. Written consent was obtained from all the participants (or their caregivers) after having been informed of the nature of the study they would take part in. Lastly, the participants were informed that they had the option to terminate the experiment at any time they wished, without having to provide justifications for their decision, and that their withdrawal would not affect, in any way, their medical treatment.

### 2.3. Statistical Analysis

The distribution of the data was checked by use of the Shapiro–Wilk test. Baseline group characteristics are presented as absolute values (n) for categorical variables and as means {with the respective standard deviations (SD)} or median {with the respective interquartile range (IQR)} for the continuous variables, in the case of a normal distribution. For the continuous variables, the differences between the baseline characteristics (in each group, P-PPC and C-BCT) were estimated by *t*-tests, in the case of normal distribution. Otherwise, the Mann–Whitney U test was applied. The comparisons for the categorical variables were performed by applying the Pearson’s chi-square test.

In order to evaluate the cognitive performance progress in each group separately over the period of 15 weeks, we compared the paired mean difference of the two assessments (baseline vs. endpoint) fo reach group, using the paired sample *t*-test and the Wilcoxon signed-rank test in the cases of normal and non-normal distribution, respectively.

The main effect of the intervention was estimated using ANCOVA or Quade’s non-parametric ANCOVA [42] in the cases of normal and non-normal distribution, respectively. In these analyses, the mean cognitive changes between the two assessments were sequentially used as the dependent continuous variables, whereas the main effects of the interventions were compared after adjusting for the covariate of the corresponding baseline scores, as previously described [42]. The level of significance was set at 0.05 for all the analyses. All statistical calculations were performed using the SPSS for Windows (version 26) statistical software (SPSS INC., Chicago, IL, USA).

## 3. Results

We did not find significant differences between the P-PCT and the C-BCT groups for demographic characteristics and baseline neuropsychological scores prior to the interventions. The demographic characteristics and clinical scores of both groups at baseline are presented in Table 1 and Table 2.

We found that the neuropsychological performance of the P-PCT group improved significantly on recall (*p* = 0.009), delayed memory (*p* = 0.024), verbal fluency (*p* = 0.019), TMT-A (*p* < 001), TMT-B (*p* < 001), IADL (*p* < 001), and MoCA (*p* = 0.029) compared to their baseline performance. The neuropsychological performance of the C-BCT group improved significantly on delayed memory (*p*= 0.009), BNT (*p* = 0.004), DFT (*p* = 0.014), DBT (*p* = 0.015), TMT-B (*p* = 0.016) compared to their baseline performance. The baseline scores of the neuropsychological tests of the C-BCT and P-PCT groups are shown in Table 3.

When comparing the two groups after the intervention (P-PCT vs. C-BCT), we noticed that the P-PCT group performed significantly better on the recall (*p* = 0.008) and IADL (*p* = 0.024) tests when compared to the C-BCT group. The C-BCT group was significantly superior on the BNT (*p* < 0.001), DFT (*p* < 0.001), and DBT (*p* < 0.001) test when compared to the P-PCT group. The comparison between the P-PCT and C-BCT groups is depicted in Table 4.

## 4. Discussion

Considering the limited pharmacological therapies available for AD, non-pharmacological interventions were designed to improve patients’ cognitive deficit using music [30], physical exercise [29] computer- and paper-and-pencil-based cognitive training, and, more recently, transcranial magnetic stimulation in combination with cognitive remediation [31,43]. The present preliminary study aimed to investigate whether C-BCT or P-PCT was more beneficial in patients with mild AD and if the interventions had the potential to transfer the gained benefits on cognitive and language abilities to everyday functional activities. The results of our study demonstrated that the different cognitive training methods had a differing impact on the cognitive and language abilities in early-stage AD patients. In particular, when the training was applied by computer-based means, it demonstrated significant improvements on delayed, working, and short-term memory and on language abilities. On the other hand, when the training was applied by paper and pencil, the results demonstrated significant improvements in the domains of global cognitive function, delayed memory, recall, attention, language, visuospatial abilities, executive function, and functional abilities.

The results of the present study agree with previously conducted studies using computer-aided training, that also reported seminal effects after the intervention in their training groups on delayed memory [10,16,17,18], working memory [10,13,18,26],verbal fluency, and executive function [10,12,15]. Contrary to our study and the one conducted by Cavallo et al. (2016) [18], in which no improvement was found on general cognitive ability and IADL, several studies reported beneficial effects on general cognitive ability [10,11,12,13,14,15] and on quality of life [14,15,16]. Lastly, the studies by Cavallo et al. (2016) and Viola et al. (2011) mentioned stability or no improvements in the domains of attention, processing speed, and global cognition. These findings agree with our results [16,18].

On the other hand, many studies investigated the beneficial impact of cognitive training using the P-PCT method. Our results of a beneficial outcome for the P-PCT intervention are in agreement with many previous studies regarding daily functional abilities [14,19,25,26,44], global cognitive profile [22,23,26,28,45], attention [20,24,25,26,27,28,46], verbal fluency [14,23,26,27,28,44,46], delayed memory [22,24,26], executive functions [14,19,20,21,24,25,26,27,28], and recall [22,24,26,28]. On the contrary, three studies [17,22,24] found no impact of the training on patients’ language abilities.

In our study, though, no improvement was found in the domains of naming and working memory (for similar results, see also Matsuda et al. (2010), Bergamaschi et al. (2013), Kim et al. (2015) [21,23,25]). These findings challenge other studies that indicated benefits on working memory [20,27] and naming [27].

Το the best of our knowledge, only three studies [14,17,26] have till now investigated and compared the effects of the two methods (on patients with dementia, mild AD, and MCI, respectively). In more detail, the study of Tsolaki et al. (2017) demonstrated remarkable similarities with our methodology and results, with the only difference being that their sample consisted of MCI individuals. Specifically, not only their “paper–pencil” training group demonstrated similar results as regards the benefited domains (attention, global cognition, language, recall), but, more importantly, this group’s IADL, a variable of great importance when applying cognitive interventions, also improved [26]. Furthermore, both our study and Tsolaki et al. (2017) demonstrated improvements on working memory and attention in the “computer-based” groups [26]. Moreover, our results agree with the study of Man et al. (2012), which explored the effectiveness of a memory-targeted program, applied by computer or paper and pencil, and found beneficial effects on delayed memory, recall, and daily functional abilities in the paper-pencil group [17]. Contrary to our results, they reported that the computer training improved more cognitive domains compared to the paper-and-pencil training. The reasons for this difference are possibly that their intervention targeted memory more specifically and that they did not train or measure other domains and variables as we did. Moreover, the computer methods they used were based on virtual reality. Lastly, as far as the Lee et al. (2013) study is concerned, the number of results in this paper similar to ours is less [14]. Specifically, our results are in agreement, for the P-PCT group, on global cognition and IADL and, for C-BCT group, on delayed memory. The remaining results do not show any similarity, possibly because the intervention program was mainly targeting and measuring domains related to memory. The above finding supports the second aim of our study, in that the prospect of the cognitive benefit was transferred to everyday functioning capacity. Not only the P-PCT method positively influenced the verbal positive feedback on daily activities and functional communication but, furthermore, the IADL questionnaire improved after P-PCT. The study by Giebeland Challis (2015) [47] reported that the majority of interventions indicated improved everyday activity performance in patients with early dementia. Focusing on the individual, as opposed to a group, daily activities appeared to be an important determinant of intervention success in patients with mild dementia. On the other hand, the systematic review of Reijnders et al. (2013) [48] suggested that the issue of whether the effects of cognitive interventions generalize to improvement in everyday life activities is still unresolved and needs to be addressed more explicitly in future research.

It is worth mentioning that the beneficial effects of cognitive training programs applied by either P-PCT or C-BCT to pwAD present great similarities, despite the pros and cons of each method. As a result, the contribution of each method has not yet been clarified [49,50,51]. A possible explanation for this might be the ‘’generation gap’’ concerning technology use. Studies in the near future will surely demonstrate greater validity, because technology is rapidly advancing, and people in their middle adulthood (40–59 years) and older age (60+ years) already successfully use in all aspects of their life and work with technological utilities (computers, tablets, smartphones, etc.).

Summing up, our study’s results reveal that the cognitive training benefits noted in our study in early-AD patients differed, depending on the method of intervention. In this respect, multi-method cognitive training (using of both C-BCT and P-PCT), focused on the personalized needs of AD individuals, may possibly delay the progression of patients’ cognitive and language deterioration, while its impact could be transmitted to patients’ daily functional abilities.

Despite the importance of our findings, it is essential to mention certain limitations of the study. Firstly, the sample size was small, raising the possibility that our study may be hampered in examining the absolute effect of C-BCT versus P-PCT in patients with mild AD. Moreover, as the follow-up period was relatively short [52], we acknowledge that a longer follow-up period could provide more variable results. Moreover, the possibility of the existence of the Hawthorne effect in the intervention cannot be completely excluded [53]. According to Berthelot et al. (2019), the Hawthorne effect is the tendency of some people to work harder and perform better when they are participants in an experiment [53].

Furthermore, it is not clarified in this study why cognitive functional training utilizing similar training tasks but conducted by a computer or via pencil–paper training, differentially impacted the trained cognitive domains. Another issue that may limit the generalization of our findings is that the transference of the primary cognitive gains was limited to the P-PCT method. This finding may possibly be due to the greater psychological benefits of paper–pencil training and the development of a better therapeutic alliance compared to computerized training.

## 5. Conclusions

Taking into consideration the limited efficacy of the available pharmaceutical interventions for AD, it becomes clear that searching for alternative interventions, especially at the early stages of AD, and examining their efficiency is a matter of great importance. Our preliminary study revealed that both C-BCT and P-PCT programs have positive effects on patients’ cognition but lead to the improvement of different cognitive and language domains. Moreover, it appeared that only the P-PCT intervention had an impact on patients’ daily lives. The maintenance of cognitive training gains also remains a major issue, and a better understanding of the barriers involved, as well as the development of effective strategies to overcome them, are needed. Unquestionably, the need for large-scale neurobehavioral interventions to further clarify this issue remains a priority.

## Figures and Tables

**Table 1:** Demographic characteristics of the participants.

	Paper-Pencil Cognitive Training (P-PCT) Group (N = 10)	Computer-Based Cognitive Training (C-BCT) Group (N = 10)	*p*-Value
Sex male/female	3/7	3/7	1.00 ^a^
Age (years), mean (SD)	75.00 (5.21)	75.20 (4.73)	0.929 ^b^
Years of education, median (IQR)	9.50 (5.00)	7.50 (7.00)	1.00 ^c^
IADL (score), mean (SD)	12.60 (1.83)	13.70 (2.26)	0.248 ^b^
GDS, median, (IQR)	2.00 (2.00)	2.00 (2.00)	0.631 ^c^

Abbreviations: IQR; interquartile range, SD; standard deviation, IADL; Instrumental Activities of Daily Living, GDS; Geriatric Depression Scale. ^a^ chi-square test. ^b^ *t*-test. ^c^ Mann–Whitney U test.

**Table 2:** Neuropsychological test scores of the groups at baseline.

	P-PCT Group (N = 10)	C-BCT Group (N = 10)	*p*-Value
Recall, median, (IQR)	18.00 (4.00)	18.00 (1.00)	0.971 ^c^
Delay memory, median, (IQR)	0.00 (0.00)	0.00 (0.00)	1.00 ^c^
MoCA, mean (SD)	16.30 (1.49)	17.00 (1.49)	0.308 ^b^
Verbal fluency, mean (SD)	21.80 (6.32)	22.40 (8.23)	0.857 ^b^
BNT, mean (SD)	12.30 (1.25)	12.30 (1.49)	1.00 ^b^
DFT, median, (IQR)	4.50 (2.00)	5.00 (2.00)	0.436 ^c^
DBT, median, (IQR)	3.00 (1.00)	3.00 (1.00)	0.912 ^c^
TMT-A, mean (SD)	182.70 (55.25)	149.80 (37.65)	0.137 ^b^
TMT-B, mean (SD)	300 (0.00)	300 (0.00)	1.00 ^b^

Abbreviations: IQR; interquartile range, SD; standard deviation, BNT; Boston Naming Test, SF; Semantic Fluency, CDT; Clock Drawing Test, DSF; Digit Span Forward, DSΒ; Digit Span Backward, TMT-A; Trial Making Test A, TMT-B; Trial Making Test B, MoCA; Montreal Cognitive Assessment, P-PCT; Paper–Pencil Cognitive Training, C-BCT; Computer-Based Cognitive Training, ^b^ *t*-test, ^c^ Mann-Whitney U test.

**Table 3:** Pre- and post-assessment scores of the groups.

			P-PCT Group			C-BCT Group				
	Pre-Assesment	Post-Assesment				Pre-Assesment	Post-Assesment			
	Mean (SD)	Mean (SD)	95% Confidence Interval of the Difference	Effect Size	*p*-Value	Mean (SD)	Mean (SD)	95% Confidence Interval of the Difference	Effect Size	*p*-Value
Recall	17.00 (3.30)	18.20 (2.78)	1.20 (0.38, 2.01)	0.51	*0.009* ^b^	17.50 (1.35)	17.00 (2.00)	−0.50 (−1.52, 0.52)	0.24	0.273 ^b^
Delay memory	0.20 (0.42)	1.10 (0.73)	0.90 (0.27, 1.52)	0.50	*0.024* ^b^	0.20 (0.42)	1.30 (0.48)	1.10 (0.57, 1.62)	0.58	*0.009* ^b^
BNT	12.30 (1.25)	12.30 (0.82)	0.00 (−0.58, 0.58)	0	1.000 ^b^	12.30 (1.49)	14.20 (0.63)	1.90 (1.11, 2.68)	0.63	*0.004* ^b^
Verbal fluency	21.80 (6.32)	24.20 (6.81)	2.40 (0.48, 4.31)	0.89	*0.019* ^a^	22.40 (8.23)	23.00 (6.89)	0.60 (−2.06, 3.26)	0.16	0.622 ^a^
DFT	4.70 (1.05)	4.20 (0.78)	−0.50 (−1.40, 0.40)	0.27	0.236 ^b^	5.10 (0.87)	6.00 (0.66)	0.90 (0.37, 1.42)	0.55	*0.014* ^b^
DBT	3.30 (0.48)	3.00 (0.66)	−0.30 (−0.88, 0.28)	0.25	0.257 ^b^	3.20 (0.78)	4.20 (0.63)	1.00 (0.41, 1.58)	0.54	*0.015* ^b^
TMT-A	182.70 (55.25)	158.00 (48.25)	−24.70 (−31.94, −17.45)	2.43	*< 0.001* ^a^	149.80 (37.65)	136.50 (31.09)	−13.30 (−32.43, 5.83)	0.49	0.150 ^a^
TMT-B	300.00 (0.00)	261.50 (21.35)	−38.50 (−53.77, −23.22)	1.80	*< 0.001* ^a^	300.00 (0.00)	279.00 (22.46)	−21.00 (−37.06, −4.93)	0.93	*0.016* ^a^
IADL	12.60 (1.83)	14.60 (1.77)	2.00 (1.10, 2.89)	1.6	*0.001* ^a^	13.70 (2.26)	14.30 (2.21)	0.60 (−0.16, 1.36)	0.55	0.111 ^a^
MoCA	16.30 (1.49)	17.20 (1.22)	0.90 (0.11, 1.68)	0.81	*0.029* ^a^	17.00 (1.49)	17.00 (1.15)	0.00 (−0.47, 0.47)	0	1.000 ^a^

Abbreviations: SD; standard deviation, BNT; Boston Naming Test, SF; Semantic Fluency, CDT; Clock Drawing Test, DSF; Digit Span Forward, DSΒ; Digit Span Backward, TMT A; Trial Making Test A, TMT B; Trial Making Test B, MoCA; Montreal Cognitive Assessment, P-PCT; Paper-Pencil Cognitive Training, C-BCT; Computer-Based Cognitive Training. ^a^ paired sample *t*-test (effect size is presented as Cohen’s d absolute values). ^b^ Wilcoxon signed-rank test (effect size is presented as r absolute values).

**Table 4:** Effect of the intervention in the P-PCT group compared to the C-BCT group.

	P-PCT Group (N = 10)	C-BCT Group (N = 10)			*p*-Value
	Mean (SD)	Mean (SD)	Treatment Difference	Effect Size	
Recall	1.20 (1.13)	−0.50 (1.43)	1.70 (0.48, 2.92)	0.33	*0.008* ^b^
Delay memory	0.90 (0.87)	1.10 (0.73)	−0.20 (−0.96, 0.56)	0.028	0.476 ^b^
BNT	0.00 (0.81)	1.90 (1.10)	−1.90 (−2.81, −0.98)	0.83	*<0.001* ^b^
Verbal fluency	2.40 (2.67)	0.60 (3.71)	1.80 (−1.26, 4.86)	0.08	0.237 ^a^
DFT	−0.50 (1.26)	0.90 (0.73)	−1.40 (−2.39, −0.40)	0.58	*<0.001* ^b^
DBT	−0.30 (0.82)	1.00 (0.81)	−1.30 (−2.07, −0.53)	0.50	*<0.001* ^b^
TMT-A	−24.70 (10.13)	−13.30 (26.75)	−11.40 (−31.19, 8.39)	0.01	0.637 ^a^
TMT-B	−38.50 (21.35)	−21.00 (22.46)	−17.50 (−38.09, 3.09)	0.15	0.091 ^a^
IADL	2.00 (1.24)	0.60 (1.07)	1.40 (0.30, 2.49)	0.25	*0.024* ^b^
MoCA	0.90 (1.10)	0.00 (0.66)	0.90 (0.03, 1.76)	0.15	0.086 ^b^

Abbreviations: SD; standard deviation, BNT; Boston Naming Test, SF; Semantic Fluency, CDT; Clock Drawing Test, DSF; Digit Span Forward, DSΒ; Digit Span Backward, TMT-A; Trial Making Test A, TMT- B; Trial Making Test B, MoCA; Montreal Cognitive Assessment. ^a^ ANCOVA. ^b^ Non-parametric (Quade’s) ANCOVA. Effect size is presented as Partial Eta squared.

**Figure A1:**
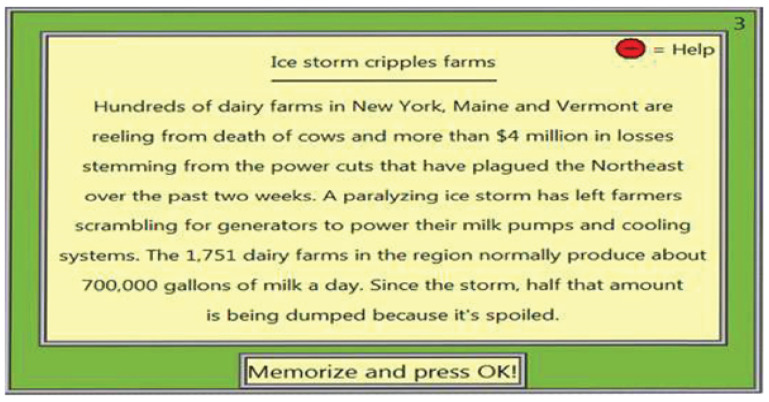
Example of verbal episodic memory.

**Figure A2:**
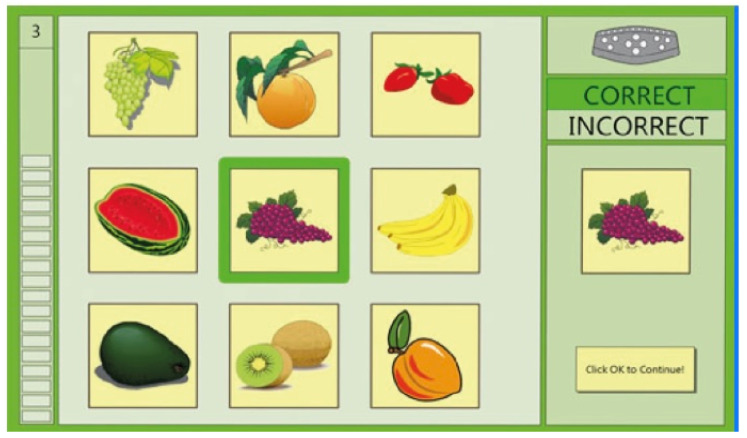
Example of attention.

## Data Availability

The data are available from the corresponding author on reasonable request.

## Appendix A

### Detailed Description of the RehaCom

To train verbal episodic memory, short stories are presented on the screen. The patients have to memorize as many details as possible (such as numbers, dates, events, names, etc.). The learning phase ends by pressing the OK button. Then, after that, the task taker should answer questions concerning the content of each story. More than 80 different-level stories are available (see Figure A1).

For the training of attention, one target image is presented separately every time at the edge of the screen and is compared to the images in the main frame of the screen. The goal is the identification and the indication of the matching picture to the target picture. (See Figure A2).

The software is available commercially in Greece, by Ostracon.

For more details about this product, see the Ostracon website at https://www.ostracon.gr/product/proionta-ana-katigoria/noitiki-endynamosi/hasomed-rehacom-logismiko-gnostikis-apokatastasis/ (accessed on 20 January 2023).
